# Association between hearing loss, tinnitus, and chronic kidney disease: the NHANES 2015–2018

**DOI:** 10.3389/fmed.2024.1426609

**Published:** 2024-07-19

**Authors:** Yihong Zou, Xiaona Tang, Kelang Rao, Yanghong Zhong, Xushan Chen, Yuyan Liang, Ying Pi

**Affiliations:** Seventh Clinical Medical College, Guangzhou University of Chinese Medicine, Shenzhen, China

**Keywords:** chronic kidney disease, hearing loss, tinnitus, association, NHANES

## Abstract

**Background:**

Previous studies suggested that chronic kidney disease (CKD) might contribute to hearing loss and tinnitus. Patients with CKD are often at risk of sudden onset hearing loss and tinnitus; however, few epidemiological investigations have been conducted on this topic. The purpose of this investigation was to analyze the correlation between hearing loss, tinnitus, and CKD based on information obtained from the National Health and Nutrition Examination Survey (NHANES).

**Methods:**

Using data from the NHANES 2015–2018, a cross-sectional analysis was conducted, which included 5,131 participants, and used multivariate logistic regression analyses and subgroup analysis to investigate the association between hearing loss, tinnitus, and CKD.

**Results:**

CKD was associated independently with hearing loss, with the CKD group being more at risk of hearing loss than the non-CKD group [age-adjusted 95% confidence interval (CI): 1.54 (1.31–1.8), *p* < 0.001]; multivariable-adjusted 95% CI: 1.31 (1.1–1.55), *p* = 0.002. Multifactorial logistic regression analysis did not find an association between CKD and tinnitus, however, further subgroup analyses showed a relationship in some populations.

**Conclusion:**

The results suggested that CKD is associated with hearing loss and tinnitus. The complexity of the relationship between CKD and hearing loss requires further research.

## 1 Introduction

In recent years, kidney disease has become a public health problem worldwide, with more than half a million people with end-stage renal disease in the USA alone (1). Chronic Kidney Disease (CKD) is a major disease that seriously jeopardizes human health in contemporary times, characterized by high morbidity, high disability, high medical costs and high susceptibility to other diseases in combination. The Global Burden of Disease study measured the number of years lived with disabilities and found that hearing loss ranked as the fourth leading cause of disability worldwide, and about two-thirds of people aged 70 or over have hearing loss (1–3). Chronic kidney disease (CKD) can be complicated by otolaryngological problems, such as epistaxis, taste disorders, and dry mouth, with the most common complication being damage to the auditory vestibular system (4); Several studies have shown that there is a strong connection between the ear and the kidney during development, and that both are dependent on proper organization patterns during embryogenesis, as well as the proper formation of shared structural elements, such as ciliated cells and basement membrane components (5). Chronic kidney disease (CKD) can be complicated by otolaryngological problems, such as epistaxis, taste disorders, and dry mouth, with the most common complication being damage to the auditory vestibular system (4); Clinically, it is often characterized by problems such as hearing loss and tinnitus, for example, Alport syndrome is a cause of chronic kidney disease, and it usually accompanies hearing loss. Hemodialysis for kidney failure has long been linked to hearing loss, there is also some hearing loss associated with inappropriate use of medications, such as aminoglycosides. And hearing loss is a common disease, when they coexist with chronic kidney disease, causing serious suffering and medical burdens for patients. Hearing loss will affect the quality of life, work and study, and can seriously bring about interpersonal communication barriers. Long-term hearing loss will also have some psychological impact on people, affecting the ability to think and express themselves, leading to anxiety and depression. The complication of tinnitus and hearing loss in patients with kidney disease can lead to further deterioration of patients’ quality of life and seriously affect their physical and mental health. However, little attention has been paid to the relationship between CKD and hearing loss, despite being reported as far back as 1927 (6).

The kidneys are the most critical organ for removing toxic chemicals from the body; when the kidneys are damaged, filtration is reduced and toxic compounds accumulate in the bloodstream, which could impair the function of the inner ear (4). Ikeda et al. found that the etiology of hearing loss mainly includes metabolic disorders, such as uremia, electrolyte imbalance, or endocrine abnormalities (7). Related studies have shown that hypertension, diabetes, the use of ototoxic drugs, electrolyte disorders, and hemodialysis treatment are associated with hearing loss in patients with CKD (8–10). Using a guinea pig model, CKD was shown to be dominated by sensorineural hearing loss (SNHL) and high-frequency hearing loss (11, 12). Moreover, the worse the renal function and the older the patient, the higher the risk of hearing loss (6, 13).

Tinnitus and hearing loss are closely related and can affect each other, and both of them can affect the patient’s daily life, leading to a decline in the patient’s quality of life and inducing emotions such as depression (14). Despite the attention of clinicians, there is no approved treatment for tinnitus. Therefore, hearing loss and tinnitus secondary to CKD require more research to develop methods of prevention and treatment, aiming to enhance the patients’ quality of life.

The relationship between hearing impairment, tinnitus, and CKD has been reported in a number of studies; however, they were mostly small observational studies and case reports, and there is still a lack of comprehensive cross-sectional studies to assess the relationship between them. We conducted a large population-based cross-sectional survey to examine the relationship between CKD and hearing loss and tinnitus. We also analyzed the severity of CKD and hearing loss and performed subgroup analyses to explore the relationship between CKD and hearing in different populations. Therefore, the purpose of this study was to analyze the association between CKD, hearing loss, and tinnitus using data from the National Health and Nutrition Examination Survey (NHANES) from 2015 to 2018.

## 2 Materials and methods

### 2.1 Study design and population

The NHANES is a large-scale cross-sectional program of studies conducted by the National Center for Health Statistics (NCHS), which is used to evaluate the health and nutritional well-being of both adults and children residing in the USA. The NHANES program has been approved by the NCHS Ethics Committee and all participants gave informed consent. We collected participants’ data on demographics, socioeconomic status, laboratory information, dietary habits, and physiology. The survey was conducted from a multistage stratified sample of selected districts, neighborhoods, households, etc. Participants were interviewed by professionals to collect questionnaires, physically examined at mobile screening centers, and their laboratory tests such as blood and urine specimens were collected. In this study, these data were used to evaluate the correlation between hearing loss, tinnitus, and the risk of CKD. We included a total of 19,225 participants, leaving 11,288 after excluding those younger than 20 years of age. They all participated in the 2015–2016 and 2017–2018 NHANES study cycles, answered The Audiometry questionnaire (AUQ 191) about hearing and had complete hearing test results. We then left 5,630 participants after removing missing values for the main variables, and for missing covariates, 773 individuals were interpolated after using multiple interpolation, ultimately resulting in 5,131 participants being included in this study.

### 2.2 The diagnosis of CKD

The definition of CKD was established according to the KDIGO 2021 guidelines (15). The estimated glomerular filtration rate (eGFR) was calculated using the Chronic Kidney Disease Epidemiology Collaboration (CKD-EPI) equation, and age, sex, and black versus non-black race were included in the eGFR estimate. In NHANES, measurement of creatinine using a recognized high performance liquid chromatography (HPLC) method, determination of urinary albumin using solid-phase fluorescent immunoassay (FIA). Patients with CKD were identified based on an eGFR lower than 59 mL/min/1.73m^2^ or an albumin-to-creatinine ratio (ACR) higher than 30 mg/g. Participants with an eGFR ≥60 mL/min/1.73 m^2^ and without albuminuria were considered to be free from CKD (15).

### 2.3 Audiometric measurements and definition of hearing loss

NHANES examined the hearing loss and tinnitus among participants aged ≥20 years old. Subjects who self-reported their hearing status through the NHANES questionnaire were considered for analysis, and the reliability of self-reported hearing loss has been previously confirmed (16). When the score was 3, the interviewee was considered to have mild hearing loss, when the score was 4 indicates moderate hearing loss, when the score was 5 means a lot of hearing loss, and when the score is 6, it indicates deafness. Subjects answered the question, “In the past 12 months, have they been bothered by tinnitus, ringing or buzzing in the ears that lasted 5 min or more?” who answered “Yes” were considered to have a history of tinnitus (17).

### 2.4 Other clinical characteristics

From the NHANES survey, we included age, sex, race, education level, marital status, poverty income ratio, body mass index (BMI), smoking, depression, and cardiovascular disease. Race was categorized into six groups, including Mexican American, other Hispanic, non-Hispanic White, non-Hispanic Black, non-Hispanic Asian, and other race. Educational attainment was categorized as below high school, high school, and college or higher. Marital status was categorized as ‘living with a partner or married’ and ‘single, or divorced, or widowed.’ Family income to poverty ratios were classified into three categories: below 1.30, between 1.31 and 3.50, and above 3.50. A higher ratio suggested a better family economic status (18).

BMI was calculated by dividing weight in kilograms by height in meters squared (kg/m^2^) and categorized as <25.0 and ≥ 25.0 kg/m^2^ (19). Smoking status was determined by answering yes to “at least 100 cigarettes smoked in a lifetime” and whether or not they currently smoke (20). Depressive status was assessed using the Patient Health Questionnaire (PHQ-9), and when the PHQ-9 score was ≥10, the respondents were considered to be suffering from depression (19). Participants answering “yes” to having any of congestive heart failure, coronary heart disease, angina pectoris, heart attack, or stroke were considered to have cardiovascular disease.

### 2.5 Statistical analysis

For all statistical analyses, the means with the standard deviation (mean ± SD) are used to present continuous variables, while numbers or percentages are used to express categorical variables. The age, race, sex, education level, marital status, poverty income ratio, BMI, smoking, depression, and cardiovascular disease were considered as covariates. Table 1 shows the baseline characteristics comparing CKD and non-CKD patients; for categorical variables we used the χ^2^ test, and for continuous variables (age) we used the t-test. Adjusted multivariate logistic regression models were used to assess the association between hearing loss, tinnitus, and the prevalence of CKD, expressed as OR and 95% confidence interval (95% CI). We constructed four multivariate regression models to examine the associations among hearing loss, tinnitus, and CKD history (Tables 2, 3). In model 1, we adjusted for age. In model 2, we included age, sex, and race. In model 3 adjusted for all the factors in model 2 plus education level, marital status, and poverty income ratio. In model 4, we adjusted for all the factors in model 3 plus BMI, smoking, depression, and cardiovascular disease. Furthermore, to investigate potential variations in the associations across different populations, we conducted subgroup and interaction analyses based on age (using 45 years old as the cut-off to divide the participants into two groups), sex, race, BMI, and smoking habits (Tables 4, 5) (21).

**Table 1:** Characteristics of the study population, according to CKD history (*n* = 5,131).

CKD	Total	No history	History	*P*
Participants, *n* (%)	5,131	4,142 (80.7)	989 (19.3)	
Sex, *n* (%)				0.01
Male	2,472 (48.2)	1959 (47.3)	513 (51.9)	
Female	2,659 (51.8)	2,183 (52.7)	476 (48.1)	
Age (years), Mean ± SD	49.7 ± 17.6	46.6 ± 16.5	62.9 ± 16.0	< 0.001
Race/ethnicity, *n* (%)				< 0.001
Mexican American	856 (16.7)	726 (17.5)	130 (13.1)	
Other Hispanic	657 (12.8)	570 (13.8)	87 (8.8)	
White	1747 (34.0)	1,375 (33.2)	372 (37.6)	
Black	1,058 (20.6)	766 (18.5)	292 (29.5)	
Asian	618 (12.0)	548 (13.2)	70 (7.1)	
Other	195 (3.8)	157 (3.8)	38 (3.8)	
Education level, *n* (%)				< 0.001
Below high school	1,147 (22.4)	888 (21.4)	259 (26.2)	
Completed high school	1,137 (22.2)	887 (21.4)	250 (25.3)	
Beyond high school	2,847 (55.5)	2,367 (57.1)	480 (48.5)	
Marital status, *n* (%)				< 0.001
Married or living with partner	3,152 (61.4)	2,601 (62.8)	551 (55.7)	
Single, divorced, or widowed	1979 (38.6)	1,541 (37.2)	438 (44.3)	
Poverty income ratio, *n* (%)				0.3
Poverty income ratio ≤ 1	1,054 (20.5)	839 (20.3)	215 (21.7)	
Poverty income ratio > 1	4,077 (79.5)	3,303 (79.7)	774 (78.3)	
BMI, *n* (%)				< 0.001
BMI < 25	1,405 (27.4)	1,188 (28.7)	217 (21.9)	
BMI ≥ 25	3,726 (72.6)	2,954 (71.3)	772 (78.1)	
Hearing loss, *n* (%)				< 0.001
No	3,953 (77.0)	3,313 (80)	640 (64.7)	
Yes	1,178 (23.0)	829 (20)	349 (35.3)	
Tinnitus, *n* (%)				0.032
Yes	830 (16.2)	643 (15.5)	187 (18.9)	
No	4,299 (83.8)	3,497 (84.4)	802 (81.1)	
Smoking, *n* (%)				< 0.001
Yes	2,153 (42.0)	1,662 (40.1)	491 (49.6)	
No	2,978 (58.0)	2,480 (59.9)	498 (50.4)	
Congestive heart failure, *n* (%)				< 0.001
Yes	164 (3.2)	64 (1.5)	100 (10.1)	
No	4,967 (96.8)	4,078 (98.5)	889 (89.9)	
Coronary heart disease, *n* (%)				< 0.001
Yes	213 (4.2)	107 (2.6)	106 (10.7)	
No	4,918 (95.8)	4,035 (97.4)	883 (89.3)	
Angina, *n* (%)				< 0.001
Yes	136 (2.7)	73 (1.8)	63 (6.4)	
No	4,995 (97.3)	4,068 (98.2)	926 (93.6)	
Heart attack, *n* (%)				< 0.001
Yes	205 (4.0)	109 (2.6)	96 (9.7)	
No	4,926 (96.0)	4,033 (97.4)	893 (90.3)	
Stroke, *n* (%)				< 0.001
Yes	206 (4.0)	109 (2.6)	97 (9.8)	
No	4,925 (96.0)	4,033 (97.4)	892 (90.2)	
Depression, *n* (%)				< 0.001
PHQ-9 < 9	4,618 (90.0)	3,758 (90.7)	860 (87)	
PHQ-9 ≥ 10	513 (10.0)	384 (9.3)	129 (13)	

**Table 2:** Multivariable-adjusted odds ratio (OR) [with the 95% confidence interval (CI)] of the relationship between CKD prevalence and hearing loss.

	Model 1		Model 2		Model 3		Model 4	
	OR (95% CI) *p* value		OR (95% CI) *p* value		OR (95% CI) *p* value		OR (95% CI) *p* value	
With CKD	1.54 (1.31 ~ 1.8)	<0.001	1.54 (1.31 ~ 1.81)	<0.001	1.49 (1.26 ~ 1.76)	<0.001	1.31 (1.1 ~ 1.55)	0.002

Model 1: Adjusted for age; Model 2: Adjusted for age, sex, race; Model 3: Adjusted for all the factors in Model 2 plus education level, marital status, poverty income ratio; Model 4: Adjusted for all the factors in Model 3 plus BMI, smoking, depression, and cardiovascular disease.

**Table 3:** Multivariable-adjusted odds ratio (OR) [with the 95% confidence interval (CI)] of the relationship between CKD prevalence and tinnitus.

	Model 1		Model 2		Model 3		Model 4	
	OR (95% CI) *p* value		OR (95% CI) *p* value		OR (95% CI) *p* value		OR (95% CI) *p* value	
With CKD	1 (0.83 ~ 1.2)	0.972	1.02 (0.84 ~ 1.23)	0.837	1.04 (0.86 ~ 1.26)	0.664	1.19 (0.97 ~ 1.45)	0.093

Model 1: Adjusted for age; Model 2: Adjusted for age, sex, race; Model 3: Adjusted for all the factors in Model 2 plus education level, marital status, poverty income ratio; Model 4: Adjusted for all the factors in Model 3 plus BMI, smoking, depression, and cardiovascular disease.

**Table 4:** The association between the prevalence of CKD and hearing loss according to logistic regression analysis in subgroups stratified by age, sex, race, BMI, and smoking.

Subgroups analysis	Unadjusted, OR (95% CI)	*p* value	Adjusted, OR (95% CI)	*p* value	*P* for interaction
Gender					0.370
Male	2.01 (1.63 ~ 2.47)	<0.001	0.94 (0.72 ~ 1.21)	0.626	
Female	2.34 (1.87 ~ 2.92)	<0.001	1.03 (0.8 ~ 1.34)	0.799	
Age					0.770
Age < 45	0.96 (0.55 ~ 1.67)	0.882	0.89 (0.5 ~ 1.59)	0.7	
Age ≥ 45	1.61 (1.37 ~ 1.91)	<0.001	0.98 (0.81 ~ 1.2)	0.856	
Race					0.351
Mexican American	1.93 (1.27 ~ 2.92)	0.002	0.99 (0.61 ~ 1.61)	0.963	
Other Hispanic	1.83 (1.1 ~ 3.05)	0.021	1.03 (0.58 ~ 1.84)	0.91	
White	2.68 (2.12 ~ 3.39)	<0.001	1.16 (0.88 ~ 1.53)	0.284	
Black	1.97 (1.39 ~ 2.78)	<0.001	0.91 (0.6 ~ 1.38)	0.656	
Asian	2.2 (1.15 ~ 4.2)	0.017	0.89 (0.42 ~ 1.91)	0.764	
Other Hispanic	1.12 (0.5 ~ 2.51)	0.786	0.18 (0.06 ~ 0.55)	0.003	
Smoking					0.086
Yes	1.76 (1.42 ~ 2.18)	<0.001	0.85 (0.66 ~ 1.1)	0.225	
No	2.51 (2.03 ~ 3.12)	<0.001	1.1 (0.85 ~ 1.44)	0.457	
BMI					0.077
BMI < 25 kg/m^2^	3.23 (2.35 ~ 4.44)	<0.001	1.19 (0.79 ~ 1.77)	0.407	
BMI ≥ 25 kg/m^2^	1.91 (1.61 ~ 2.26)	<0.001	0.92 (0.75 ~ 1.13)	0.445	

Adjusted variables: age, race, sex, education level, marital status, poverty-to-income ratio, BMI, smoking, depression, and cardiovascular disease.

**Table 5:** The association between the prevalence of CKD and tinnitus according to logistic regression analysis in subgroups stratified by age, sex, race, BMI, and smoking.

Subgroups analysis	Unadjusted, OR (95% CI)	*P* value	Adjusted, OR (95% CI)	*P* value	*P* for interaction
Gender					0.301
Male	0.73 (0.58 ~ 0.93)	0.011	1.06 (0.8 ~ 1.41)	0.68	
Female	0.88 (0.67 ~ 1.16)	0.382	1.59 (1.17 ~ 2.16)	0.003	
Age					0.408
Age < 45	1 (0.58 ~ 1.71)	0.995	1.12 (0.64 ~ 1.97)	0.691	
Age ≥ 45	1 (0.82 ~ 1.21)	0.972	1.29 (1.03 ~ 1.6)	0.027	
Race					0.578
Mexican American	0.89 (0.56 ~ 1.41)	0.613	1.51 (0.9 ~ 2.54)	0.123	
Other Hispanic	0.72 (0.4 ~ 1.31)	0.286	1.34 (0.69 ~ 2.62)	0.39	
White	0.71 (0.54 ~ 0.93)	0.014	1.1 (0.81 ~ 1.5)	0.547	
Black	1.05 (0.7 ~ 1.59)	0.809	1.65 (1.02 ~ 2.68)	0.041	
Asian	1.09 (0.38 ~ 3.17)	0.872	1.68 (0.53 ~ 5.35)	0.382	
Other Hispanic	0.55 (0.25 ~ 1.22)	0.142	1.16 (0.44 ~ 3.05)	0.769	
Smoking					0.092
Yes	0.74 (0.58 ~ 0.94)	0.015	0.99 (0.75 ~ 1.32)	0.972	
No	0.93 (0.7 ~ 1.22)	0.59	1.84 (1.34 ~ 2.51)	<0.001	
BMI					0.330
BMI < 25 kg/m^2^	0.59 (0.4 ~ 0.88)	0.01	0.88 (0.56 ~ 1.41)	0.604	
BMI ≥ 25 kg/m^2^	0.87 (0.71 ~ 1.07)	0.19	1.47 (1.17 ~ 1.85)	0.001	

Adjusted variables: age, race, sex, education level, marital status, poverty-to-income ratio, BMI, smoking, depression, and cardiovascular disease.

We use multiple imputation to count for missing data on depression, calculated Cronbach’s coefficient to assess the internal reliability of the imputed data, and selected the data set with the largest Cronbach’s coefficient for analysis (22). The predictive mean matching (PMM) model was chosen to determine the interpolation values and a total of 773 cases of missing values were interpolated. Missing data to account for age, race, gender, educational attainment, marital status, poverty-to-income ratio, BMI, smoking, depression, and cardiovascular disease.

In this study, all analyses were performed using SPSS (version 25.0; IBM Corporation, Armonk, NY, USA) and Empower Stats 165 software (X&Y solutions, Inc., Boston, MA, USA).^1^ All statistical tests in the study were conducted using a two-sided approach. *p* < 0.05 was considered as statistically significant.

## 3 Results

The baseline demographics of the participants are shown in Table 1. The non-CKD group comprised 4,142 (80.7%) individuals, and the CKD group comprised 989 (19.3%) individuals. Among the participants, the mean ages of the two groups in the study were 46.6 ± 16.5 and 62.9 ± 16.0 years, respectively. Among the CKD group, 51.9% were male, 78.1% had a high BMI, and 21.7% were living in a family with income at or below poverty. Compared with those in the non-CKD group, individuals in the CKD group were more likely to be male, older, and suffer from depression and cardiovascular disease.

Tables 2, 3 show the relationship between hearing loss or tinnitus, respectively, and the incidence of CKD. Multivariate logistic regression analysis found a statistically significant correlation between hearing loss and CKD in all individuals. Individuals with CKD were more likely to have concurrent hearing impairment than those without. After adjusting for all covariates factors that could influence the results, hearing loss was still positively associated with CKD in the population aged ≥20 years old (95% confidence interval (CI) = 1.1–1.55, *p* = 0.002); however, no such significance was found among tinnitus sufferers.

We also performed subgroup analyses and interactions based on population characteristics, including age, sex, race, BMI, and smoking to analyze the association between hearing loss, tinnitus, and the incidence of CKD, as shown as in Tables 4, 5. Adjusted variables: age, race, sex, education level, marital status, poverty-to-income ratio, BMI, smoking, depression, and cardiovascular disease. The subgroup analyses and interaction test showed that the association between hearing loss and CKD was not statistically different between strata, indicating that age, gender, race, BMI, and smoking did not have a significant effect on this association (*p* > 0.05; Table 4). However, tinnitus was significantly associated with CKD in subgroups stratified by age, sex, race, BMI, and smoking (*p* < 0.05), specifically, among those aged ≥45 years, the multivariate OR (95% CI) in the tinnitus group was (1.03–1.6), and in the female group the multivariate (1.17–2.16) in the female group, (1.34–2.51) in the non-smoking group, and (1.17–1.85) in the BMI ≥ 25 kg/m^2^ group. And the interaction showed that the association between tinnitus, hearing loss, and CKD was unmodified in selected subgroups of age, sex, race, BMI, and smoking (*p* for interaction >0.05).

## 4 Discussion

In this cross-sectional study of nationally representative data from the USA, the aim of the study was to explore the relationship between hearing loss, tinnitus, and CKD. Multivariate logistic regression analyses revealed that hearing loss was associated with CKD, and this association remained after adjusting for potential confounders. In contrast, no relationship was found between tinnitus and CKD, which contradicts the results of previous studies (22, 23). This might be because the mechanisms linking tinnitus and CKD are more complex and their relationship cannot be captured by a cross--sectional study design.

Our observed correlation between CKD and hearing loss is consistent with the results of previous studies, showing that CKD is significantly associated with increased hearing loss (6, 23, 24), particularly sensorineural deafness (4, 13, 25). Vilayur et al. explored the relationship between eGFR and hearing loss in a cross-sectional study, and found the highest prevalence of hearing loss in subjects with eGFR ≤45 mL/min/1.73 m^2^ (6); however, that study only included people over the age of 49. Jong-Yeup et al. found that compared with those without CKD, middle-aged patients with CKD had a higher incidence of sensorineural deafness and Ménière’s disease (13). However, that study did not obtain patient-specific information such as eGFR, and SSNHL and Ménière’s disease was diagnosed according to diagnostic codes, which did not guarantee accuracy. Gatland et al. measured hearing thresholds in 31 patients receiving hemodialysis and found that patients with chronic renal failure had a higher prevalence of low and high frequency hearing loss (8); however, the included sample size was limited. These studies reported similar patterns in the relationship between CKD and hearing loss; however, most of them are small sized studies. By contrast, we used the NHANES database for our analyses, which has wide range and large number of participants, uses standard questionnaires and accurate laboratory tests, and addresses several limitations of the previous literature.

There is a study suggest no correlation between CKD and hearing. Vilayur et al. measured serum creatinine (SCr) in 1,843 individuals without hearing loss and performed hearing tests at baseline and at 5, 10, and 15 years of follow-up, and found no significant correlation between declining creatinine-based eGFR and hearing loss, which might have resulted from differences in study populations and definitions of SCr measurements (26).

Although the exact mechanism of auditory dysfunction in patients with CKD is unknown, there are several hypotheses. The kidney and the cochlea, two seemingly unrelated organs, show anatomical and physiological similarities (8). Anatomically, Quick et al. used immunochemical and immunohistochemical tools to compare the kidney and cochlea sidewalls of rats and guinea pigs, and the results confirmed a striking similarity between the cochlea and the kidney (27). Sensory hearing loss predominates in patients with CKD, and sensorineural deafness is associated with cochlear and vascular stripe damage (2). The vascular stripe of the inner ear and the glomerular basement membrane share the same antigenic properties and both are immunologically relevant (28). Physiologically, the cochlear Na+, K+- ATPase plays a key role in maintaining the cochlear cation gradient; if significant ATPase activity is present, a large number of mitochondria are found, a feature that is evident in vascular striae and renal tubular cells (27). It was also suggested that inhibition of this enzyme system might be associated with uremic inner ear dysfunction (29). Moreover, CKD electrolyte disturbances might also contribute to hearing loss (25).

Hearing loss is an irreversible injury that can seriously affect patients’ quality of life. There is evidence for a relationship between CKD and hearing loss; however, there is a lack of standard clinical guidelines or expert consensus. Early clinical manifestations of CKD in patients are not significant, thus paying attention to hearing changes can prompt patients to seek a clinical consultation. Such a physical examination might detect early renal lesions, which would allow timely to prevention of the occurrence of hearing loss, involving early hearing assessment and testing, and timely feedback to the doctor. Healthcare professionals should then employ certain treatments in a timely manner to slow down the progression of the disease, such as acupuncture or fitting the patients wear hearing aids. In terms of treatment, care should be taken when prescribing ototoxic drugs, such as furosemide and aminoglycoside antibiotics, and drugs that are protective of the kidneys and hearing can be chosen. In future scientific research, the physiopathologic relationship between the kidneys and the ear can be further explored to seek better treatments and drug development, and comprehensive treatment to provide patients with a better quality of life rather than simply treating the disease. In conclusion, the relationship between hearing impairment and CKD requires ongoing research to discover the detailed relationships, with the aim of developing novel treatments.

The strengths of this study are: 1. The sample size was large and standardized baseline laboratory data and questionnaires were used; 2. We performed a stratified analysis, and the results showed that gender, age, weight, and smoking status had an effect on the tinnitus status of CKD patients. Nevertheless, given the limited sample size of tinnitus we investigated, this finding should be interpreted with caution and need further prospective studies; 3. We analyzed both hearing and tinnitus status in the CKD population, which makes this study more comprehensive compared to studying only hearing status.

The limitations of this study are: 1. This study is not longitudinal and causal inferences cannot be made; 2. This study was not weighted; 3. Due to the limitations of the database, we did not include all the influencing factors related to hearing and CKD. Nonetheless, our findings suggest a relationship between CKD and hearing loss, which has some clinical significance. In the future, a larger and more detailed sample size is expected, thus further elucidating the relationship between decreased renal function and hearing loss; 4. In our study, we defined tinnitus as “In the past 12 months, have they been bothered by tinnitus, ringing or buzzing in the ears that lasted 5 min or more?” who answered “Yes” were considered to have a history of tinnitus. If tinnitus sufferers were defined in this way, it would be expected that the prevalence of tinnitus would be higher in the population, and since CKD does not cause most tinnitus, more data on tinnitus in these patients is therefore needed to better represent the relationship between tinnitus and CKD.

## 5 Conclusion

Our findings suggest a significant association between hearing loss, tinnitus and CKD. We hypothesized that people with CKD are more at risk for hearing loss and tinnitus. However, the results do not establish a causal relationship and further extensive prospective studies are needed.

## Data availability statement

The datasets presented in this study can be found in online repositories. The names of the repository/repositories and accession number(s) can be found at: https://www.cdc.gov/nchs/nhanes/index.htm.

## Author contributions

YiZ: Conceptualization, Data curation, Investigation, Methodology, Software, Writing – original draft. XT: Data curation, Writing – review & editing. KR: Writing – original draft. YaZ: Writing – original draft. XC: Writing – review & editing. YL: Conceptualization, Writing – review & editing. YP: Conceptualization, Writing – review & editing.

## Footnotes

^1^ www.empowerstats.com
